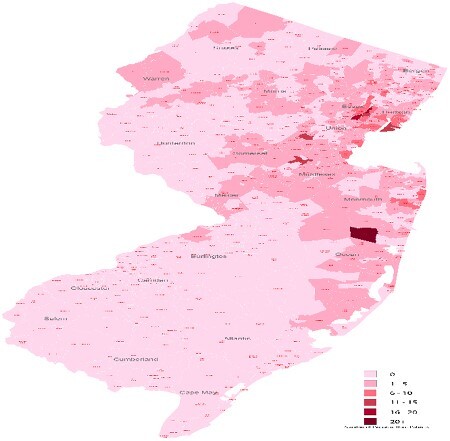# 733 Pediatric Burns: A Single Institution Retrospective Review of Incidence, Etiology, and Outcomes in 685 Patients

**DOI:** 10.1093/jbcr/irae036.276

**Published:** 2024-04-17

**Authors:** Bilal M Chaudhry, Subin Lee, Jonathan Pineda, Michael A Marano, Robin A Lee, Cherry Song, Christina J Lee

**Affiliations:** Cooperman Barnabas Medical Center, Toms River, NJ; Cooperman Barnabas Medical Center, Livingston, NJ; Cooperman Barnabas Medical Center, Verona, NJ; Cooperman Barnabas Medical Center, Toms River, NJ; Cooperman Barnabas Medical Center, Livingston, NJ; Cooperman Barnabas Medical Center, Verona, NJ; Cooperman Barnabas Medical Center, Toms River, NJ; Cooperman Barnabas Medical Center, Livingston, NJ; Cooperman Barnabas Medical Center, Verona, NJ; Cooperman Barnabas Medical Center, Toms River, NJ; Cooperman Barnabas Medical Center, Livingston, NJ; Cooperman Barnabas Medical Center, Verona, NJ; Cooperman Barnabas Medical Center, Toms River, NJ; Cooperman Barnabas Medical Center, Livingston, NJ; Cooperman Barnabas Medical Center, Verona, NJ; Cooperman Barnabas Medical Center, Toms River, NJ; Cooperman Barnabas Medical Center, Livingston, NJ; Cooperman Barnabas Medical Center, Verona, NJ; Cooperman Barnabas Medical Center, Toms River, NJ; Cooperman Barnabas Medical Center, Livingston, NJ; Cooperman Barnabas Medical Center, Verona, NJ

## Abstract

**Introduction:**

Nearly 300 children die from burn injuries every year, and over 100,000 are admitted to a hospital or treated in an emergency department in the United States. Although largely preventable, burns remain a leading cause of morbidity in the pediatric population globally. This study assesses all pediatric burn admissions to a State Wide Certified Burn Treatment Center to evaluate trends in demographics, burn incidence, and cause across different age groups.

**Methods:**

Demographic and clinical data were collected on all pediatric burn admissions during an 8 year period (2015-2022). These patients were stratified by age into "age 0 to 6," "age 7 to 12," and "age 13 to 18." Data were analyzed using standard statistical methodology. A state wide map was created allocating all patients into their respective zip codes to aid in targeting pediatric burn prevention programs.

**Results:**

A total of 685 burn patients under age 18 were treated between 2015 and 2022. A total of 511 (74.6%) patients were ages 0 to 6, 89 (12.9%) were 7 to 12, and 85 (12.4%) were age 13 to 18. A total of 362 (52.8%) were male and 323 (47.2%) were female (male: female ratio of 1.12:1). Hispanics had the highest burn admissions across all age groups (35.8%), followed by African-Americans (23.6%). Caucasian teenagers formed the largest part (41%) of the teenage admissions. Mean TBSA burned was 8.9%, with torso being the most common site (68%). Scald burns constituted the majority of cases (83.4%, n = 571), with 70% attributable to hot liquids related to cooking, including coffee, tea or soup. In the 0-6 age group scald burns were the overwhelming cause (92.6%). In the teenage group, flame burns were the dominant cause (47.1%). Overall mean length of stay was 10.9 days for all patients. A total of 159 patients (23.2%) required an ICU admission. Seventeen patients (2.6 %) required a ventilator. Overall mortality was 0.4% (n = 3).

**Conclusions:**

The majority of pediatric burn admissions were caused by scald burns, ages 0-6 were the most vulnerable in this regard. Flame burns predominated in the teenage population. Mean TBSA burned was 8.9% with the torso being the most likely to be involved. Mean length of stay was 11 days, 23% required ICU and 2.6% required a ventilator. Mortality was 0.4%.

**Applicability of Research to Practice:**

The results of this study emphasize the need to target etiology specific burn prevention programs against scald burns in the very young (0-6yrs) and flame burns in the teenage population. Geographic depiction aids in highlighting the more vulnerable counties in our state.